# RETRACTION: The Effect of Mesenchymal Stem Cell‐Derived Exosomes and miR17‐5p Inhibitor on Multicellular Liver Fibrosis Microtissue

**DOI:** 10.1155/sci/9804587

**Published:** 2026-04-17

**Authors:** Stem Cells International

RETRACTION: F. Sani, M.S. Zomorrod, N. Azarpira, and M. Soleimani, “The Effect of Mesenchymal Stem Cell‐Derived Exosomes and miR17‐5p Inhibitor on Multicellular Liver Fibrosis Microtissue,” *Stem Cells International*, vol. 2023 (2023) https://doi.org/10.1155/2023/8836452.

The above article, published online on 4 August 2023 in Wiley Online Library (wileyonlinelibrary.com), has been retracted following the agreement of the journal’s Chief Editor Ren‐Ke Li; and John Wiley & Sons Ltd.

The retraction has been agreed following an investigation of the issues raised by *Elizabeth M Bik* on PubPeer [1], which highlighted concerns related to Figure 7a in the manuscript.

More specifically, the panel “LX2+anti‐miR” contains multiple unexpectedly overlapping features.

The authors provided the raw data supporting the conclusions of the article, however the data were not sufficient to address the concerns. As a result of this investigation, the results of the article are no longer considered reliable.

The authors disagree with this retraction.

## References

[1] Bik E. M. , The Effect of Mesenchymal Stem Cell-Derived Exosomes and miR17-5p Inhibitor on Multicellular Liver Fibrosis Microtissue, *PubPeer*, October 2025. https://pubpeer.com/publications/183A0342DC82A27CD1C3ED1D6E4361. 10.1155/2023/8836452 PMC10421706 37576406

